# Length of sick leave – Why not ask the sick-listed? Sick-listed individuals predict their length of sick leave more accurately than professionals

**DOI:** 10.1186/1471-2458-4-46

**Published:** 2004-10-12

**Authors:** Nils Fleten, Roar Johnsen, Olav Helge Førde

**Affiliations:** 1 Department of Community Medicine, University of Tromsø, Tromsø, N-9037, Norway; 2 Department of Community Medicine and General Practice, Norwegian University of Science and Technology, Trondheim, Norway

## Abstract

**Background:**

The knowledge of factors accurately predicting the long lasting sick leaves is sparse, but information on medical condition is believed to be necessary to identify persons at risk. Based on the current practice, with identifying sick-listed individuals at risk of long-lasting sick leaves, the objectives of this study were to inquire the diagnostic accuracy of length of sick leaves predicted in the Norwegian National Insurance Offices, and to compare their predictions with the self-predictions of the sick-listed.

**Methods:**

Based on medical certificates, two National Insurance medical consultants and two National Insurance officers predicted, at day 14, the length of sick leave in 993 consecutive cases of sick leave, resulting from musculoskeletal or mental disorders, in this 1-year follow-up study. Two months later they reassessed 322 cases based on extended medical certificates. Self-predictions were obtained in 152 sick-listed subjects when their sick leave passed 14 days. Diagnostic accuracy of the predictions was analysed by ROC area, sensitivity, specificity, likelihood ratio, and positive predictive value was included in the analyses of predictive validity.

**Results:**

The sick-listed identified sick leave lasting 12 weeks or longer with an ROC area of 80.9% (95% CI 73.7–86.8), while the corresponding estimates for medical consultants and officers had ROC areas of 55.6% (95% CI 45.6–65.6%) and 56.0% (95% CI 46.6–65.4%), respectively. The predictions of sick-listed males were significantly better than those of female subjects, and older subjects predicted somewhat better than younger subjects. Neither formal medical competence, nor additional medical information, noticeably improved the diagnostic accuracy based on medical certificates.

**Conclusion:**

This study demonstrates that the accuracy of a prognosis based on medical documentation in sickness absence forms, is lower than that of one based on direct communication with the sick-listed themselves.

## Background

The increasing rate of sick leave experienced in most Western countries challenges insurance companies, employers, and public authorities to identify measures to reduce burdens at the individual, workplace and societal levels.

To reduce the expenses of sick leave and the risk of expulsion from work, the Norwegian government introduced legislation in 1993 that anticipated early and more vigorous interventions of the Norwegian National Insurance Scheme [1]. The Norwegian Public Report no. 27 [2], 2000, underscored the importance of early intervention by the National Insurance Offices (NIOs). A major challenge for the NIOs is to identify newly sick-listed individuals at risk of prolonged sick leave, and who are therefore potential candidates for rehabilitating interventions.

The selection process is currently based on information in medical sickness certificates supplied by access to the register of previous sickness benefits. A medical sickness certificate (Sickness Certificate 1; SC1) is required if sick leave exceeds 3 days, and after 8 weeks an extended medical certificate is mandatory (Sickness Certificate 2; SC2) [3]. In addition to diagnosis and certified period, the majority of SC1s contain information on the occupation and employee, whereas information on chronic disease, previous sick leave episodes, prognosis and comments are more scattered. SC2s include updated medical information on work ability, planned diagnostics and treatments, and on the prognosis. The value of this information as a guideline for selective intervention has, however, never been established, either as an indicator of potential prolonged absence, or as an indicator of the need for occupational or vocational rehabilitation [4].

Based on the current practice with identifying sick-listed individuals at risk of long-lasting sick leaves, the objectives of this study were to inquire diagnostic accuracy of predictions within the NIOs, and to compare their predictions with the self-predictions of the sick-listed.

## Methods

**Figure 1:**
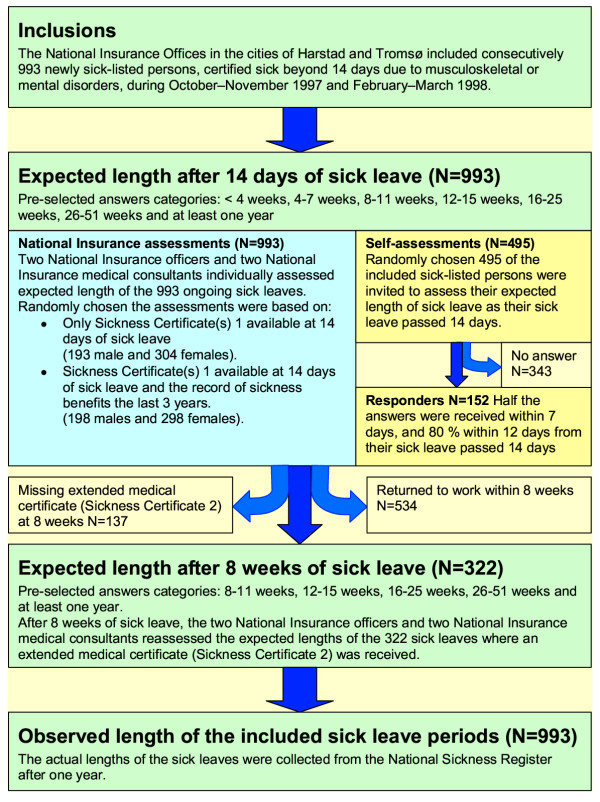
**Flow-chart. **Flow-chart of inclusion, and the different assessments of expected length, of the included sick leaves after 2 and 8 weeks of sick leave.

In October and November 1997 and March and April 1998, newly sick-listed persons with musculoskeletal or mental disorders (ICPC, L- and P- diagnoses) [5] were included consecutively if they were certified sick for longer than 2 weeks (Figure 1). Five hundred persons were included in each period. The study took place in the cities of Tromsø and Harstad in Northern Norway. The total length of sickness benefits was registered during the following year in the National Sickness Benefit Register. Missing data on the length of sick leave reduced the number of included subjects to 993. The mean ages of these 391 men and 602 women were 41.4 and 39.7 years, respectively. Musculoskeletal disorders were the main reason for sick leaves (83% of the cases).

A total of 495 randomly selected persons received a questionnaire on the expected length of their ongoing sick leave period. The answer categories were: less than 4 weeks, 4 to 7 weeks, 8 to 11 weeks, 12 to 15 weeks, 16 to 25 weeks, 26 to 51 weeks, and at least 1 year. Some 152 persons (30.7%), called the responder group, returned the questionnaire with this question filled in.

Based on SC1s available after 14 days of sick leave, two NIO officers without formal medical competence, but experienced in working with sick-listed persons, and two experienced physicians working part time as insurance medical officers (NIO medical consultants), assessed the expected length in each of the 993 ongoing sick leave cases. In 496 randomly chosen cases, the NIO assessors had additional access to information on sick leave periods during the previous 3 years. Of potentially 1986 assessments in each profession, the officers and medical consultants had 18 and 25 missing assessments, respectively.

SC2s became available in 322 of the 459 cases where sick leave exceeded 8 weeks, and the NIO assessors reassessed these cases.

Reproducibility of assessments by medical consultants were analysed in 20 cases reassessed by the two NIO medical consultants, and assessed by another eight of their colleagues.

### Observed length of sick leaves

The reference standard lengths of individual sick leaves within 1 year were collected from the National Sickness Benefit Register. Sick leaves interrupted by only 1–2 days without sickness benefits, typically on weekends, were registered as a single period. The observed length of sick leave thus comprised the total period of continuous full-time or part-time absence due to sickness within 1 year.

### Statistics

The diagnostic accuracy of predicted lengths was compared on the basis of sensitivity, specificity, likelihood ratio and the area under the receiver operating characteristics curves (ROC area) [6,7]. The non-parametric standard error and 95% CI for the ROC area were calculated in SPSS-11. The ROC curve represents plots of the true-positive rate (sensitivity) and the false positive rate (1 – specificity) at the average of two consecutive categories of the assessments (>= 0 weeks, >= 4 weeks, >= 8 weeks etc). The ROC curves of the mean assessment by NIO officers and medical consultants include even intermediate points representing half categories.

The predictive validity is presented as sensitivity, specificity, positive predictive value (PPV) and likelihood ratio at different thresholds, cut-offs, in predicted length [8]. Reliability of predicted length was analysed with agreement between assessors, the kappa value [9,10].

### Approval

The Regional Ethical Committee approved the protocol, and the Norwegian Data Inspectorate licensed the necessary register of sick-listed subjects.

## Results

The mean observed continuous sickness absence was 100.8 days (median 48 days). Sick leaves in females lasted a mean of 105.1 days, compared to 94.6 days in men (medians 55 and 43 days, respectively). The mean length among persons with musculoskeletal disorders was 90.2 days in 335 males and 108.6 days in 489 females. The mean length among persons with mental disorders was 120.6 days in 56 males and 90.0 days in 113 females.

The mean length of the sick leave in the responder group was 107.4 days (95% confidence interval, CI, 88.7–126.1 days), compared to 92.4 days in the 343 non-responders. Stratified analysis revealed longer mean sick leaves among responders 40 years and younger, of 109.3 days (95% CI 81.4–134.5 days), compared to the 79.3 days (95% CI 65.6–93.1 days) in non-responders. Stratification on gender or musculoskeletal or mental disorders did not reveal any significant differences in the length of sick leave between responders and non-responders.

All assessors, including the sick-listed themselves, systematically overestimated the length of short sick leaves (lasting 4–11 weeks) and underestimated the length of long sick leaves (exceeding 16 weeks; Table 1). The proportions of sick leaves lasting longer than 8, 12 or 26 weeks did not differ significantly between the responder group and the rest.

**Table 1:** Categorical distribution of observed and predicted length of sick leave. Observed and predicted length of sick leaves in seven categories for all participants (n = 993) compared to the responder group (n= 152). The assessments of National Insurance medical consultants and officers are grouped according to proportions of persons predicted in each category.

	All participants Proportion according to			Responder group Proportion according to			
		
Length of sick leave categories	Observed length %	Assessed by medical consultants % 95% CI	Assessed by officers % 95% CI	Observed length % 95% CI	Assessed by medical consultants % 95% CI	Assessed by officers % 95% CI	Assessed by sick-listed % 95% CI
< 4 weeks	31.7	27.6 25.7–29.7	18.9 17.2–20.7	29.6 22.5–37.5	32.2 27.0–37.8	20.9 16.4–25.9	25.0 18.3–32.7
4–7 weeks	22.0	41.8 39.6–44.0	36.8 34.7–39.0	25.0 18.3–32.7	40.9 35.3–46.7	33.4 28.1–39.1	36.2 28.6–44.4
8–11 weeks	12.9	20.3 18.6–22.2	25.4 23.5–27.4	7.2 3.7–12.6	18.3 14.1–23.1	26.8 21.9–32.2	15.1 9.8–21.8
12–15 weeks	6.2	7.0 5.9–8.3	13.7 12.2–15.3	3.9 1.5–8.4	6.3 3.8–9.7	13.6 9.9–18.0	10.5 6.1–16.5
16–25 weeks	9.3	1.0 0.6–1.6	1.9 1.3–2.6	13.2 8.2–19.6	0.7 0.1–2.4	2.6 1.2–5.2	5.9 2.7–10.9
26–51 weeks	6.8	0.7 0.4–1.2	0.7 0.4–1.2	9.9 5.6–15.8	0.3 0.0–1.8	0.0 0.0–1.2	1.3 0.2–4.7
>= 52 weeks	11.1	1.5 1.0–2.1	2.5 1.9–3.3	11.2 6.7–17.3	1.3 0.4–3.2	2.6 1.2–5.2	5.9 2.7–10.9

### Receiver operating characteristics of prediction

**Figure 2:**
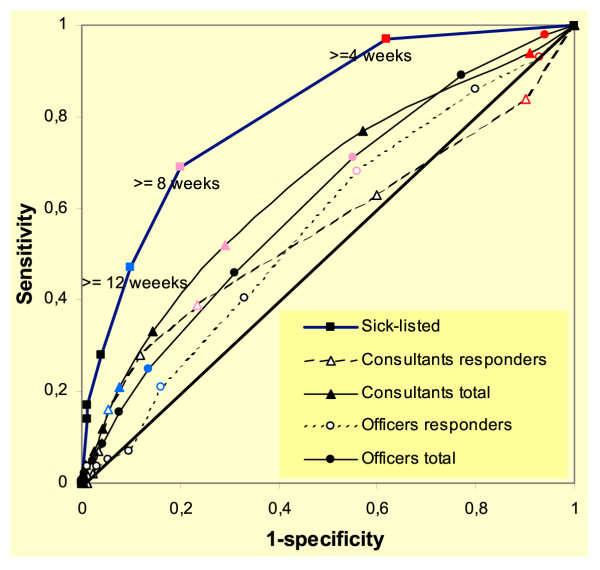
**ROC curves of identifying sick leaves lasting at least 12 weeks. **The ROC curve of ability to identify sick leaves lasting at least 12 weeks, plotted at the average of two consecutive categories, in length predicted by sick-listed (n = 152), and mean length predicted by National Insurance officers and medical consultants in the responder group (n = 149, 150) and for all the data (n= 972, 975). The points representing cut-offs in predicted length >= 4 weeks (red), >= 8 weeks (pink) and >= 12 weeks (blue) are identified.

The sick-listed subjects predicted sick leaves equal to or longer than 12 weeks more accurately than the NIO medical consultants and officers, as shown by the ROC curve in Figure 2. The differences in ROC area between responders and non-responders were most marked among younger subjects and in females (Table 2). Generally, the length of sick leave was predicted more accurately in older subjects than in younger subjects, and better in males than in females. Access to past history of sick leaves improved the ROC area of NIO consultants from 60.6% (95% CI 51.3–69.9%) to 75.4% (95% CI 68.2–82.6%) in male sick-listed, but did not improve the ROC area in assessments of female sick-listed.

**Table 2:** ROC area of identifying sick leaves lasting at least 12 weeks. The ability to identify sick leaves lasting at least 12 weeks in the responder group (n = 152) and in all participants (N = 993), presented as ROC area, calculated from length of sick leave predicted by sick-listed, and mean length predicted by National Insurance medical consultants and officers. The range of the individual National Insurance ROC areas is presented for all participants.

			Medical consultants			Officers		
				
		Self-assessed Responders *n *= 152	Responders *n *= 149	All participants *n *= 972		Responders *n *= 150	All participants *n *= 975	
		
Sick-listed	*n*	ROC area	ROC area	ROC area	Range individual	ROC area	ROC area	Range individual
	*N*	95% CI	95% CI	95% CI	ROC area	95% CI	95% CI	ROC area
All	152	80.9	55.6	64.6	59.6–64.2	56.0	61.4	55.6–65.6
	993	73.7–86.8	45.6–65.6	60.8–68.3		46.6–65.4	57.7–65.1	

17–40 years of age	78 508	76.4 65.6–87.2	43.0 28.8–57.2	57.2 51.7–62.8	54.5–57.8	48.9 35.4–62.5	57.4 52.0–62.9	51.9–57.8
							
41–67 years of age	74 485	85.7 77.2–94.2	68.3 54.8–81.8	70.7 65.8–75.6	63.4–70.1	62.5 49.4–75.6	65.1 60.1–70.1	56.1–73.4
								
Males	56	90.9	63.0	68.7	62.8–68.3	59.6	63.6	56.5–71.8
	391	83.4–98.4	47.3–78.7	62.8–74.6		44.3–74.9	57.5–69.8	

Females	96	74.7	50.9	62.0	57.7–61.5	54.0	60.3	52.9–61.8
	602	64.8–84.7	38.1–63.8	57.2–66.8		42.0–65.9	55.6–64.9	

**Figure 3:**
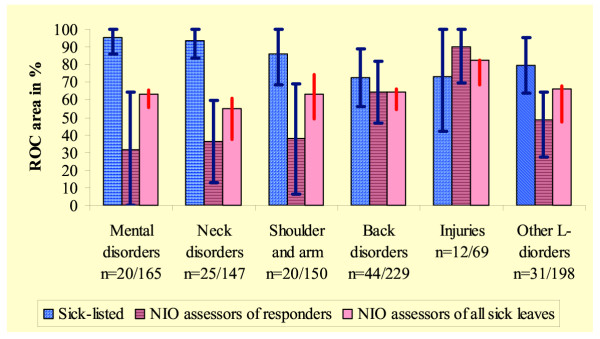
**ROC area in different diagnostic groups. **ROC area representing ability to identify sick leaves 12 weeks or longer in different diagnostic groups, calculated on length predicted by sick-listed, and mean of lengths predicted by NIO assessors. The ROC area are presented with blue bars of 95% CI in the responder group (n = 152/), and red bars without horizontal lines between upper and lower individual ROC area of the NIO assessors for all sick leaves (n = /958).

Changing the observed length to be identified from 12 weeks to 8 or 26 weeks did not significantly change the diagnostic accuracy as assessed by the ROC area. The sick-listed identified sick leaves lasting 8 weeks or longer with a ROC area of 79.5% (95% CI 72.2–85.6%), and sick leaves lasting 26 weeks or longer with a ROC area of 75.5% (95% CI 67.9–82.1%). Sick-listed persons with mental disorders or with neck, or shoulder and arm disorders, were most accurate in their assessment (Figure 3). This was in contrast to NIO assessors, who demonstrated the lowest predictive ability in these diagnostic groups, particularly in responders. The impact on diagnostic accuracy of knowing the occupation was small.

### Sensitivity, specificity, predictive value and likelihood ratio

The sick-listed subjects predicted their sick leaves with higher sensitivity and PPV than the NIO assessors (Tables 3, 4). Male sick-listed predicted sick leaves lasting at least 12 weeks with a sensitivity of 0.82% (95% CI 0.60–0.95) and a PPV of 0.78 (95% CI 0.56–0.93) using predicted length of at least 8 weeks. The corresponding sensitivity and PPV of female sick-listed were both 0.61 (95% CI 0.44–0.77).

**Table 3:** Predictive validity – identifying sick leaves lasting at least 12 weeks. Predictive validity of identifying sick leaves that lasted at least 12 weeks, using 8 weeks as the cut-off in length as predicted by the sick-listed, medical consultants and officers. The prediction based on the Sickness Certificate 2 (SC2) used a cut-off in predicted length of at least 12 weeks. Sensitivity, specificity, PPV, and likelihood ratio data for NIO assessors are presented as means with 95% CI.

Predicted length	*n *assessments	Sensitivity (95% CI)	Specificity (95% CI)	Likelihood ratio (95% CI)	PPV^1 ^(95% CI)	PPV adjusted to prevalence 33.4% (95% CI)
Sick-listed	152	0.69 (0.56–0.84)	0.80 (0.70–0.87)	3.4 (1.9–6.2)	0.68 (0.54–0.79)	0.63 (0.49–0.76)
Medical consultants Responder group	301	0.35 (0.26–0.44)	0.78 (0.71–0.84)	1.6 (1.0–2.5)	0.49 (0.38–0.61)	0.44 (0.33–0.56)
Medical consultants All participants	1961	0.42 (0.38–0.45)	0.75 (0.73–0.77)	1.7 (1.4–1.9)	0.45 (0.41–0.49)	
Officers Responder group	302	0.53 (0.44–0.62)	0.59 (0.51–0.66)	1.3 (0.9–1.8)	0.44 (0.36–0.53)	0.39 (0.31–0.48)
Officers All participants	1968	0.53 (0.49–0.57)	0.60 (0.58–0.63)	1.3 (1.2–1.5)	0.40 (0.37–0.43)	
Medical consultants SC2	637	0.85 (0.82–0.88)	0.44 (0.36–0.52)	1.5 (1.2–1.9)	0.82 (0.79–0.86)	0.43 (0.39–0.48)
Officers SC2	636	0.88 (0.86–0.91)	0.33 (0.26–0.41)	1.3 (1.1–1.7)	0.80 (0.77–0.84)	0.40 (0.35–0.44)

^1^The prevalence of sick leaves lasting at least 12 weeks was 38.2% in the responder group (*n *= 152), 33.4% for all participants (*n *= 993), and 72.1% in the SC2 group (*n*= 322).

**Table 4:** Predictive validity – identifying sick leaves lasting at least 26 weeks. Predictive validity of the ability to identify sick leaves lasting at least 26 weeks, using 8, 12 or 26 weeks, as cut-offs in length as predicted by the sick-listed, medical consultants or officers. Sensitivity, specificity, PPV and likelihood ratio data for NIO assessors are presented as means for length predicted on Sickness Certificates 1 and Sickness Certificates 2 (SC2).

Predicted length	*n *assess-ments	Sensitivity (95% CI)	Specificity (95% CI)	Likelihood ratio (95% CI)	PPV^1 ^(95% CI)	PPV adjusted to prevalence 17.9%(95% CI)
Sick-listed >= 8 weeks	152	0.69 (0.50–0.84)	0.69 (0.60–0.77)	2.2 (1.3–3.9)	0.37 (0.25–0.51)	0.33 (0.21–0.47)
Sick-listed >= 12 weeks	152	0.50 (0.32–0.68)	0.83 (0.75–0.90)	3.0 (1.5–6.1)	0.44 (0.28–0.62)	0.40 (0.24–0.58)
Sick-listed >= 26 weeks	152	0.28 (0.14–0.47)	0.98 (0.94–1.00)	16.9 (3.5–160)	0.82 (0. 48–0.98)	0.78 (0.44–0.95)
Consultants >= 8 weeks	1961	0.44 (0.39–0.49)	0.72 (0.70–0.74)	1.6 (1.3–1.9)	0.25 (0.22–0.29)	
Consultants >= 12 weeks	1961	0.20 (0.16–0.24)	0.92 (0.90–0.93)	2.4 (1.8–3.2)	0.35 (0.28–0.41)	
Consultants >= 26 weeks	1961	0.07 (0.04–0.10)	0.99 (0.98–0.99)	2.8 (1.5–5.4)	0.54 (0.38–0.69)	
Officers >= 8 weeks	1968	0.55 (0.50–0.60)	0.58 (0.56–0.61)	1.3 (1.1–1.5)	0.22 19.3–24.9	
Officers >= 12 weeks	1968	0.26 (0.21–0.31)	0.83 (0.81–0.85)	1.6 (1.3–2.0)	0.25 (0.20–0.29)	
Officers >= 26 weeks	1968	0.06 (0.04–0.09)	0.98 (0.97–0.98)	1.5 (0.9–2.6)	0.34 (0.23–0.48)	
SC2						
Consultants >= 12 weeks	637	0.89 (0.86–0.93)	0.29 (0.25–0.34)	1.3 (1.1–1.5)	0.44 (0.40–0.48)	0.22 (0.18–0.25)
Consultants >= 26 weeks	637	0.24 (0.19–0.29)	0.96 (0.93–0.98)	5.9 (3.4–11.0)	0.79 (0.68–0.87)	0.56 (0.41–0.70)
Officers >= 12 weeks	636	0.89 (0.85–0.93)	0.21 (0.17–0.25)	1.1 (0.9–1.3)	0.41 (0.37–0.45)	0.20 (0.16–0.23)
Officers >= 26 weeks	636	0.28 (0.22–0.34)	0.90 (0.87–0.93)	2.8 (1.9–4.3)	0.64 (0.54–0.73)	0.38 (0.28–0.49)

^1^The prevalence of sick leaves lasting at least 26 weeks was 21.1% in the responder group (*n *= 152), 17.9% for all the data (*n *= 993), and 38.5% in the SC2 group (*n *= 322).

Duration of at least 8 weeks was the preferable cut-off in predicted length, to identify sick leaves lasting at least 12 weeks (Table 3). A predicted length of at least 12 weeks reduced the sensitivity in all the data to 0.17 in medical consultants and 0.25 in officers. The corresponding improvement in PPV was modest, reaching 0.54 in medical consultants and 0.45 in officers. Using a predicted length of at least 4 weeks would have markedly reduced the specificity (Figure 2).

The sensitivity of identifying sick leaves lasting at least 26 weeks was generally low when medical consultants and officers predicted on the basis of SC1s. (Table 4). The sensitivity was improved somewhat by introducing SC2 information, but the effects on likelihood ratio and PPV if prevalence corrected, were minor.

According to the results, the effects of the different predictive strategies can be illustrated by considering a program designed to intervene in all cases where the subject is expected to be sick-listed for more than 12 weeks at 14 days of sick leave. Out of every 1000 sick-listed persons, 333 will be sick-listed for more than 12 weeks according to the prevalence in this study. The random selection of 333 persons will include 111 true positives, while 333 persons selected by officers will include 133 of the 333 persons that will be sick-listed at least 12 weeks. The evaluation of 1000 sick-listed individuals thus increases the number of true positives by 22 in a selection of 333 sick-listed persons. The alternative strategy of asking the sick-listed themselves will include 210 true positives in a selection of 333 persons.

### Reliability and reproducibility of the predicted length

Agreement between medical consultants in their initial prediction of sick leaves lasting at least 12 weeks, was fair, with a kappa of 0.31 (95% CI 0.20–0.43). The corresponding kappa value between officers was 0.05 (95% CI -0.05–0.14).

In the prediction of sick leaves lasting at least 12 weeks based on the SC2, agreement was moderate between medical consultants (kappa = 0.42, 95% CI 0.29–0.54) and fair between officers (kappa = 0.26, 95% CI 0.10–0.42). The corresponding agreements in the prediction of sick leaves lasting at least 26 weeks were moderate between medical consultants (kappa = 0.55, 95% CI 0.40–0.70) and fair between insurance officers (kappa = 0.31, 95% CI 0.17–0.47).

The differences in diagnostic accuracy, between the two participating medical consultants and their eight colleagues in the reproducibility group, were not significant.

## Discussion

The results of the present study question any practical value of using information in medical sickness certificates in predicting the length of sick leave, as is the current practice in Norwegian NIOs. Instead, the sick-listed themselves predicted their length of sick leaves far more accurately, but this information is not routinely sought.

### Representativeness

The officers in the present study were selected from experienced officers who had shown an interest in the field of sick leave. This might introduce a bias of overestimating the officers' general ability to predict the length of sick leaves. The performances of the two medical consultants were representative of eight of their colleagues who participated in the reproducibility part of the study. We therefore consider the diagnostic accuracy of the assessors to be representative of their professional groups, or at least not underestimated due to bias. Although the diagnostic accuracy varied within each group, the main conclusion of better predictive ability among the sick-listed, was challenged neither by comparing with the mean length predicted by assessors, nor by comparing with the best-performing NIO assessor.

The distributions of gender and diagnosis among the 993 persons included in the study were comparable with those in the National Sickness Benefits Register. The findings of longer sick leaves in women with musculoskeletal disorders, and longer sick leaves in men with mental disorders, are consistent with the Register and other studies [11-13].

The low responder rate among the sick-listed introduced a possible selection bias, although we could not identify any selection bias in gender, age, diagnosis or occupation [14]. If there was a selection towards more predictable sick leaves, this should have been reflected in the assessments of officers and medical consultants. The general trend of lower diagnostic accuracy of NIO assessors in the responder group indicates that if any selection bias contributes to the results, it is an underestimate of the self-predictive ability.

### Why did the sick-listed make better predictions?

If the lengths of sick leaves were predominantly related to loss of function caused by sickness, in line with the legislation, we would expect that the medical consultants' professional competence would favour them in predictions of the lengths of sick leaves. The differences we observed between medical consultants and officers in mean ROC area, were however minor. Furthermore, we could not demonstrate any significant differences in diagnostic accuracy between medical consultants and officers when aggregate information on disease, treatment, function related to work, and prognoses were available in the SC2. The improvement in ROC area with this aggregated information was minor, with the area just reaching 70%, which is considered borderline useful for some purposes [7]. The result is in line with Bjørndal's findings of low prognostic impact of the SC2 [15], and is supported by findings of a low predictive power of symptoms and signs in neck and shoulder disorders [16]. The better prediction of the length of sick leave by the sick-listed themselves, is supported by studies that have identified different non-disease determinants of sick leave, such as job satisfaction [17], attitudes towards pain [18], irreplaceability [19] and psychosocial work environment [20-22]. Studies identifying that at least the initial sickness certification is predominantly patient controlled [23,24] indicate the competence of the sick-listed. Self-rated health seems to be an independent predictor of return to work [17], disability pension [25] and early retirement [26]. Our findings can be interpreted as indicating that the subjective perception of sickness and work ability is more predictive of the length of sick leave, than the apparently more objective description in medical terms. The differences in predictive ability were especially significant in persons with mental and neck disorders, while the NIO assessors performed equal to the sick-listed in the more clear-cut injuries with more standardised treatment and prognosis. Mental disorders, with high prevalence in the population, and an increasing cause of absence [27], are of special interest [13]. This increasing prevalence of sick leaves indicates the presence of factors separate from the diagnosis criteria. It seems that the more clear-cut the disease and the recommended treatment, the lesser the gain in predictive ability achieved by asking the sick-listed, and vice versa. The modest gain in predictive ability caused by introducing more medical information by the inclusion of the SC2 supports this interpretation. A more complete description of symptoms and treatment does not necessarily give better prognostic information when this includes little knowledge of the consequences related to occupation, and the effects of treatment are undocumented or, at best, marginal.

### Diagnostic accuracy – practical implication

The Norwegian NIO is obliged by legislation to perform early intervention on the sick-listed in an effort to reduce the length of sick leave and the risk of expulsions from work. Limited resources and the large number of sick-listed individuals make selection desirable before any intervention is initiated. An alternative to selection on the basis of medical certificates is to communicate directly with the sick-listed themselves. This selection for intervention by NIOs might be seen as screening. The aim is to reach – at an acceptable cost – as many as possible of those that might profit from intervention. The potential individual gain by intervention will be greater when longer lasting sick leaves can be anticipated, and greater the sooner individual intervention programs are established.

The marginal predictive ability and modest agreement between NIO assessors questions the use of resources in selection based on information from medical certificates. The predictions of medical consultants tend to be better than those of officers, but not to an extent that makes it more meaningful to use medical consultants in the selection process, rather than officers.

With limited resources for intervention, it might be more cost effective to identify those whose sick listing will last longer than 26 weeks instead of 12 weeks. Based on self-reporting, eight out of ten would be true positives, and one fourth of the individuals would be reached. To reach the same number of true positives at 14 days of sick leave, the ratio of true positives would be reversed from eight out of ten, to two or three out of ten, if the selection were based on medical certificates.

In the search for tests predicting long-lasting sick leaves, such as The Örebro Musculoskeletal Pain Questionnaire [28], the present study indicates that the results of any such tests should be compared with the results of crude self-estimated length.

## Conclusions

Sick-listed individuals predicted their length of sick leave far more accurately than did NIO medical consultants and officers based on information from sickness certificates and the history of past sick leaves. The predictions of sick-listed males were better than those of females, and older persons predicted better than younger persons. The availability of more information, as through the SC2, had only a minor effect on the predictive ability of the medical consultants and officers. Neither reliability nor validity of their predictions was satisfactory.

This study demonstrates the need to re-consider the diagnostic usefulness of documentation on sickness absences, and supports a change in strategy from collecting more medical information to more direct communication with the sick-listed themselves, for effective and early interventions to prevent long sick leaves and expulsions from work.

## Competing interests

The author N.F. is part-time employed as National Insurance medical consultant.

## Authors' contributions

NF was in charge of designing and running the study, and performed most of the analyses and the writing of this manuscript. RJ actively supervised all parts of the study, and OHF contributed to planning and writing. All authors read and approved the final version of the manuscript.

## Pre-publication history

The pre-publication history for this paper can be accessed here:


